# Impact of respiratory muscle training on sleep disordered breathing in people with tetraplegia: a sub-analysis of a randomised controlled trial

**DOI:** 10.1038/s41393-026-01252-8

**Published:** 2026-08-13

**Authors:** Claire L. Boswell-Ruys, R. H. Chaminda Lewis, Rachel A. McBain, Nirupama S. Wijesuriya, Danny J. Eckert, Bonsan Bonne Lee, Simon C. Gandevia, Jane E. Butler

**Affiliations:** 1 The Kolling Institute and the School of Medical Scfiences, Faculty of Medicine and Health, The University of Sydney, Camperdown, NSW Australia; 2 University of New South Wales, Sydney, NSW Australia; 3 Prince of Wales Hospital, Randwick, NSW Australia; 4 Royal North Shore Hospital, St Leonards, NSW Australia; 5 The George Institute for Global Health, Newtown, NSW Australia; 6 FHMRI Sleep Health, Flinders University, Adelaide, SA Australia

**Keywords:** Respiratory signs and symptoms, Sleep, Spinal cord diseases

## Abstract

**Study design:**

Sub-analysis of a randomised controlled trial.

**Objectives:**

Respiratory muscle training (RMT) in people with tetraplegia yields marked improvements in inspiratory muscle strength. The present study investigated whether RMT also modifies sleep disordered breathing (SDB) and daytime sleepiness.

**Setting:**

Independent research institute in Sydney, Australia.

**Methods:**

Sixty-two adults with tetraplegia underwent six weeks of supervised RMT. The active-RMT group trained the respiratory muscles through progressive threshold loading to a mean intensity of 50% maximal inspiratory pressure whereas the sham-RMT group experienced the same training protocol, but their device had no progressive threshold load. Primary measures of SDB were obtained using level II ambulatory polysomnograms and daytime sleepiness was assessed using the Epworth Sleepiness Scale (ESS) at baseline and after 6-weeks RMT.

**Results:**

Forty-eight participants completed two home-based polysomnograms. Maximal inspiratory pressure (primary outcome) increased more after active intervention than sham, between-group difference 11.8cmH_2_O (95%CI, 5.2–18.4, *p* = 0.001). There were no between group differences for any SDB parameter; mean apnoea-hypopnea index (± SD) (active 46 ± 21 vs. 45 ± 22 events/h; sham 44 ± 25 vs. 40 ± 25 events/h; *p* = 0.553) nor ESS (active 9.5 ± 5.7 vs. 9.2 ± 6.7; sham 10.8 ± 6.2 vs. 10.1 ± 5.8; *p* = 0.952).

**Conclusions:**

Despite significant increases in inspiratory muscle strength, 6 weeks of supervised respiratory muscle training at a mean intensity of 50% maximal inspiratory pressure does not reduce SDB or daytime sleepiness in people with tetraplegia and severe SDB.

## Introduction

Sleep disordered breathing (SDB) is a common sequelae of spinal cord injury (SCI) with SDB risk factors such as obesity and male sex being more common in this population compared to people without a SCI [1]. SDB includes obstructive sleep apnoea (OSA), central sleep apnoea (CSA) and sleep-related hypoventilation disorders, and is prevalent after tetraplegia [2, 3]. In tetraplegia, severe SDB (apnoea-hypopnea index (AHI) ≥ 30) prevalence peaks at 83% at 3 months post injury with a subsequent reduction in severity after 6 months [2]. However, the prevalence regardless of severity is still much greater than the non-SCI population and is reported to range from 46–97% [3].

SDB is associated with an increase in adverse cardiovascular outcomes for people without a SCI and negatively affects physical function and rehabilitation [4]. Similarly, obesity, diabetes and cardiovascular morbidity is common after SCI [5]. Cardiovascular and respiratory disease are the leading causes of death 12 months after SCI [6]. While little is known about the long-term consequences of nocturnal hypoventilation and hypercapnia in SCI, theoretical frameworks suggest that SDB contributes to increased cardiovascular mortality in chronic tetraplegia and therefore, represents a modifiable cardiovascular risk factor [7, 8]. However, there are no data on long-term outcomes or the impact of treating SDB in this population. Additionally, excessive daytime sleepiness, disturbed and poor sleep quality are common problems in people with tetraplegia [1, 9].

The reasons behind the high prevalence of SDB in tetraplegia are likely multifactorial. While the motor and sensory innervation of pharyngeal muscles that maintain upper airway patency remain intact in tetraplegia, upper airway dilator muscle reflex responses are altered in many people with tetraplegia and sleep apnoea [10]. Other factors that affect upper airway collapsibility are also altered after SCI. Increased collapsibility of the upper airway during sleep is a driver of SDB pathogenesis both non-SCI [11] as well as in tetraplegia, where the upper airway critical closing pressure of the pharynx is higher (less negative/more collapsible) [12]. Decreased lung volume and accompanying reductions in tracheal traction can increase airway collapsibility [13]. In people with tetraplegia lung volume in supine may be reduced further in the presence of high BMI or large abdominal girth [5]. Disruption of the sympathetic control of airway mucosa and high nasal resistance may also play a role in the high prevalence of SDB in people with SCI [14].

Upper airway muscles, including genioglossus, maintain upper airway patency. People with OSA have significantly higher basal genioglossal activity that is believed to compensate for a more collapsible upper airway [15]. Activity of the genioglossus is reduced at sleep onset [16] with a greater reduction in people with OSA [17]. Genioglossus activity is modulated by afferents of upper airway mechanoreceptors [18], lung and chest wall stretch receptors [10].

SDB treatment indications in SCI are similar to the general population. The gold standard of treatment is positive airway pressure (PAP) with either continuous or bi-level nocturnal non-invasive ventilation [19]. However, adherence with PAP therapy is low in the SCI population with compliance between 23 and 50% [19]. Thus, a well-tolerated, self-administered, home-based alternative therapeutic modality such as respiratory muscle training (RMT), which would also train the upper airway muscles, has merit, yet has shown mixed results. In people without a SCI threshold inspiratory muscle training (IMT) over 12-weeks improves the Functional Outcomes of Sleep Questionnaire score and snoring (*n* = 27) [20], and a reduction in the AHI and Epworth Sleepiness Scale (ESS) of people with moderately severe OSA (*n* = 22) [21] but not in people with mild to moderate OSA after 5-weeks IMT (*n* = 9) [22]. Pilot studies in tetraplegia suggest RMT increased inspiratory muscle strength, reduced AHI and improved nocturnal oxygen saturation [23, 24]. The aim of the current study was to investigate, as part of a larger randomised controlled trial, the effect of RMT on SDB severity and daytime sleepiness in people with tetraplegia. We hypothesised that AHI would reduce after RMT due to increased inspiratory muscle strength. If the severity of SDB can be reduced with RMT, it would offer a low-cost alternative form of treatment, that may be better tolerated than PAP.

## Methods

### Study design

A planned sub-analysis of a double-blind, randomised controlled trial to compare the effect of RMT on SDB severity and daytime sleepiness. The trial was pre-registered at Australian New Zealand Clinical Trials Registry (ACTRN 12612000929808) and conducted at a single site between November 2013 and December 2016. All procedures were approved by the South-Eastern Sydney Local Health District Human Research Ethics Committee (Reference No. 12/192) and the University of New South Wales Human Research Ethics Committee (Reference No. HC13388) and complied with the Declaration of Helsinki 2013.

### Participants

Participants aged over 18 years with C3-C8 neurological level of SCI and American Spinal Injury Association Impairment Scale grades A-C were recruited from Prince of Wales Hospital, Sydney or the community as part of a larger RMT trial [25]. Sixty-two people with tetraplegia participated. Those with significant chest trauma, pneumothorax, significant co-existing respiratory or neurological condition, cognitive impairment or mechanically ventilated were excluded. Prior to enrolment, informed written consent was obtained from participants or with the aid of a family member or an independent third party if unable to sign independently.

### Randomisation and blinding

A computer-generated adaptive random allocation schedule was used to minimise differences between treatment groups (sham and active; see Boswell-Ruys et al. [25] for details). Stratification was based on, time since injury (acute (< 6months) versus chronic (> year)); motor complete versus motor incomplete SCI; and a baseline AHI (above or below 15 events/h). Participants, treating therapists, sleep technicians, polysomnography scorer and assessors were blind to allocated treatment throughout the trial.

### Intervention

Participants performed supervised RMT twice daily for six-weeks using a portable threshold-RMT device (Threshold IMT, Philips Respironics, New Jersey, USA). Five days a week, 3–5 sets of 12 inspiratory breaths, sets separated by two minutes of resting breathing, were completed. Expiratory breaths followed the same protocol. The training procedure employed a nonlinear exercise regime that was tailored to increase training intensity as strength improved. Participants commenced training at 30% baseline maximal inspiratory pressure (PImax) or maximal expiratory pressure (PEmax) which thereafter increased weekly by 10% (capped at 80% PImax or PEmax). Further details about device modifications for sham or active and RMT can be found in Boswell-Ruys et al. [25]. Participants with recently acquired tetraplegia underwent usual care in their acute hospital rehabilitation program, those with a chronic injury did not partake in any other therapy.

### Outcome measures

The primary outcome was total AHI. All sleep parameters were assessed in participants’ homes using portable level II polysomnography (Alice PDx, Philips Respironics, Murrysville, Pennsylvania, USA (nasal airflow thermistors and pressure transducers were used as surrogates to detect temperature and respiration changes; a chin strap was not applied to maintain participant safety and communication)). The parameters were scored as described by The American Academy of Sleep [26] and included apnoea and hypopnoea events per hour of sleep (AHI), central sleep apnoea events (CSA), and AHI events during rapid- and non-rapid eye movement (REM and NREM) sleep and sleep duration. The minimum and mean blood oxygen desaturation (SpO_2_) during sleep, and the percentage of time that saturation was <90% were calculated. Daytime somnolence was assessed using the Epworth Sleepiness Scale (ESS). The primary outcome in the parent trial was inspiratory muscle strength measured as PImax, with secondary outcomes of lung function; inspiratory capacity (IC), vital capacity (VC), forced vital capacity (FVC), forced expiratory volume in one second (FEV_1_), peak expiratory flow during a cough (PEFc), and maximal expiratory pressure (PEmax) all measured with a portable lung function machine (Hyp’air, Belgium) following the American Thoracic Society (ATS) guidelines.

### Statistical analysis

Data were analysed using analysis of covariance (ANCOVA) adjusted for the baseline measures. The treatment effect size was estimated by the between-group mean difference and corresponding 95% confidence intervals. The relationship between baseline or change in PImax and change in AHI and change in ESS were analysed with Pearson’s correlations. Missing data were deleted on a case-wise basis. No adjustments were made for multiple comparisons. An alpha level<0.05 was considered significant. All statistical analyses were performed with IBM SPSS Statistics (v25, IBM Corp., Armonk, NY, USA).

## Results

**Fig. 1:**
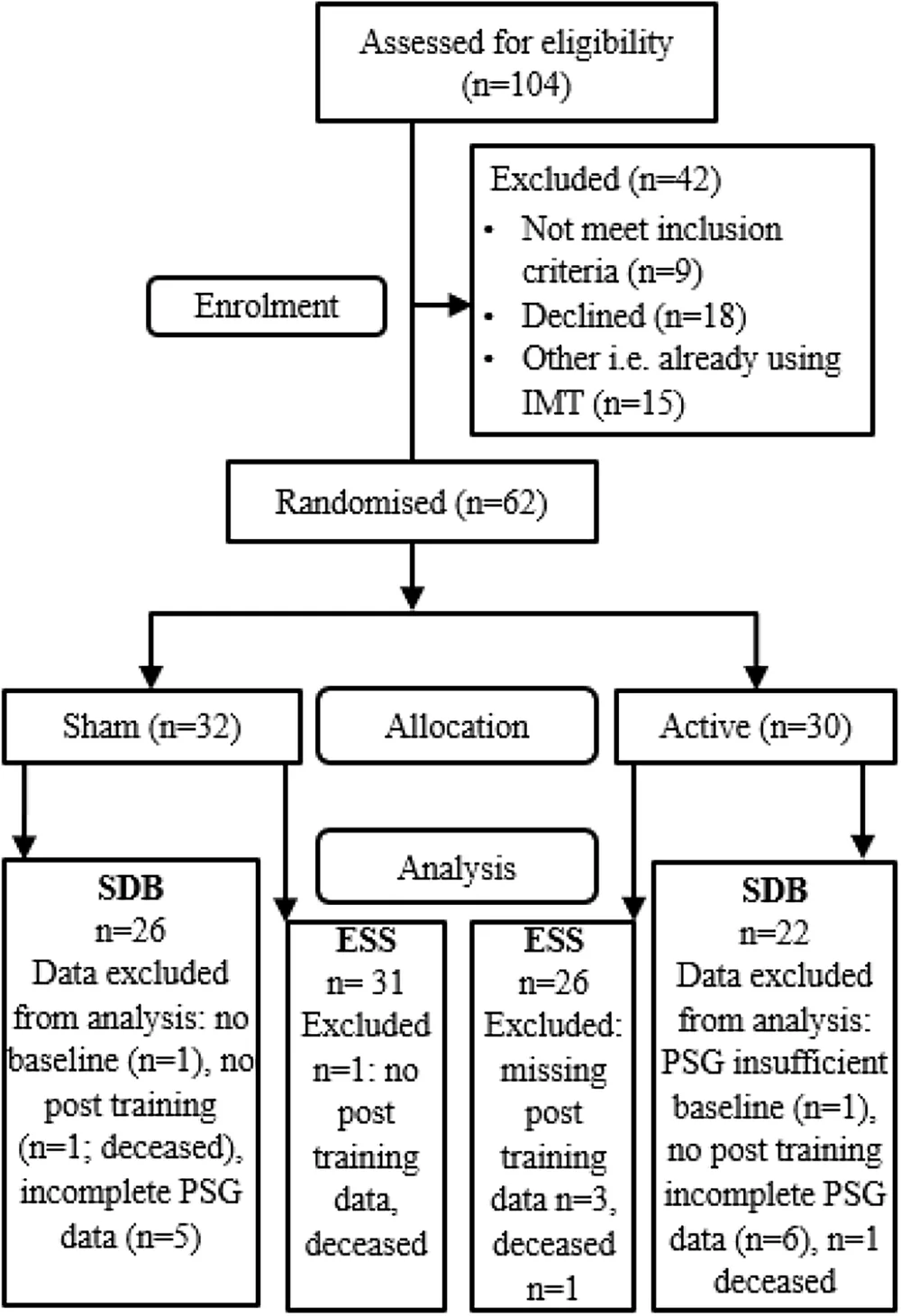
Participant flow diagram for the number (n) of participants enrolled, allocated to Sham- and Active- RMT (respiratory muscle training) groups, and included in the analyses. IMT, inspiratory muscle training; SDB, sleep disordered breathing; ESS, Epworth sleepiness scale; PSG, polysomnography.

Sixty-two people with cervical SCI were recruited; 48 participants had complete and analysable diagnostic polysomnography at baseline and after 6-weeks of supervised training, and 57 participants had complete ESS data (see Fig. 1). The RMT protocol was adhered to by all participants. Baseline characteristics (Table 1) were well matched between groups. However, no female participants were allocated to the active training group.

**Table 1:** Participant characteristics at baseline for sleep disordered breathing analysis.

	Sham RMT	Active RMT
**Number of participants**		
Total	26	22
Acute (<6 months post injury)	10	10
Chronic (>1-year post injury)	16	12
Motor Complete Injury (AIS A-B)	18	16
Motor Incomplete Injury (AIS C)	8	6
AHI < 5 events/h	0	2
AHI 5–15 events/h	2	2
AHI 16–30 events/h	4	4
AHI > 31 events/h	20	16
**Age** (years) ^a^	53.9 ± 14.9	48.4 ± 14.5
**Sex** (m:f)	22:4	22:0
**Body-mass index** (kg/m^2^) ^a^	24.4 ± 5.5	24.9 ± 5.3
**Lung function measures** ^a^		
PImax (cmH_2_O)	50.0 ± 19.2	50.6 ± 24.0
PEmax (cmH_2_O)	32.0 ± 15.1	33.0 ± 18.4
FEV_1_ (l)	1.90 ± 0.73	1.93 ± 0.79
FVC (l)	2.50 ± 0.94	2.55 ± 1.00
Vital Capacity (l)	2.59 ± 0.94	2.57 ± 0.89
Inspiratory Capacity (l)	2.06 ± 0.64	2.13 ± 0.63
PEFc (l/s)	4.74 ± 1.71	4.88 ± 1.34
**Sleep disordered breathing parameters** ^a^		
Total AHI (events/h)	43.7 ± 24.5	46.1 ± 21.3
CSA (events/h)	0.5 ± 1.5	0.4 ± 0.9
Total REM AHI (events/h)	45.8 ± 19.2	51.6 ± 30.8
Total NREM AHI (events/h)	39.4 ± 24.7	40.9 ± 21.1
Min SpO_2_ (%)	73.8 ± 15.0	75.7 ± 12.2
Time SpO_2_ < 90% (%)	8.5 ± 12.8	10.5 ± 12.5
Mean SpO_2_ (%)	93.5 ± 2.2	93.3 ± 2.1
Sleep duration (minutes)	286 ± 100	285 ± 84
**Epworth Sleepiness Scale (ESS)** ^b^		
Total (number of participants)	31	26
ESS < 11 (number of participants)	19	16
ESS > 11 (number of participants)	12	10
ESS (score/24) ^a^	10.8 ± 6.2	9.5 ± 5.7

*RMT* respiratory muscle training, *AIS* American Spinal Injury Association (ASIA) Impairment Scale, *AHI* apnoea hypopnoea index, *PImax* Maximal Inspiratory Pressure, *PEmax* maximal expiratory pressure, *FEV*_*1*_ forced expiratory volume in 1 s, *FVC* forced vital capacity, *PEFc* peak expiratory cough flow, *CSA* central sleep apnoea, *REM* rapid eye movement, *NREM* non-rapid eye movement, *Min SpO*_*2*_ minimum oxygen saturation; Time SpO_2_ < 90%, Percentage of time with oxygen saturation <90%; *Mean SpO*_*2*_ mean oxygen saturation, *ESS* Epworth sleepiness scale.

^a^means ± SD; ^b^ ESS questionnaires completed by more participants and analysed with increased sample size, however ESS comparisons to other data were performed on complete datasets only.

**Fig. 2:**
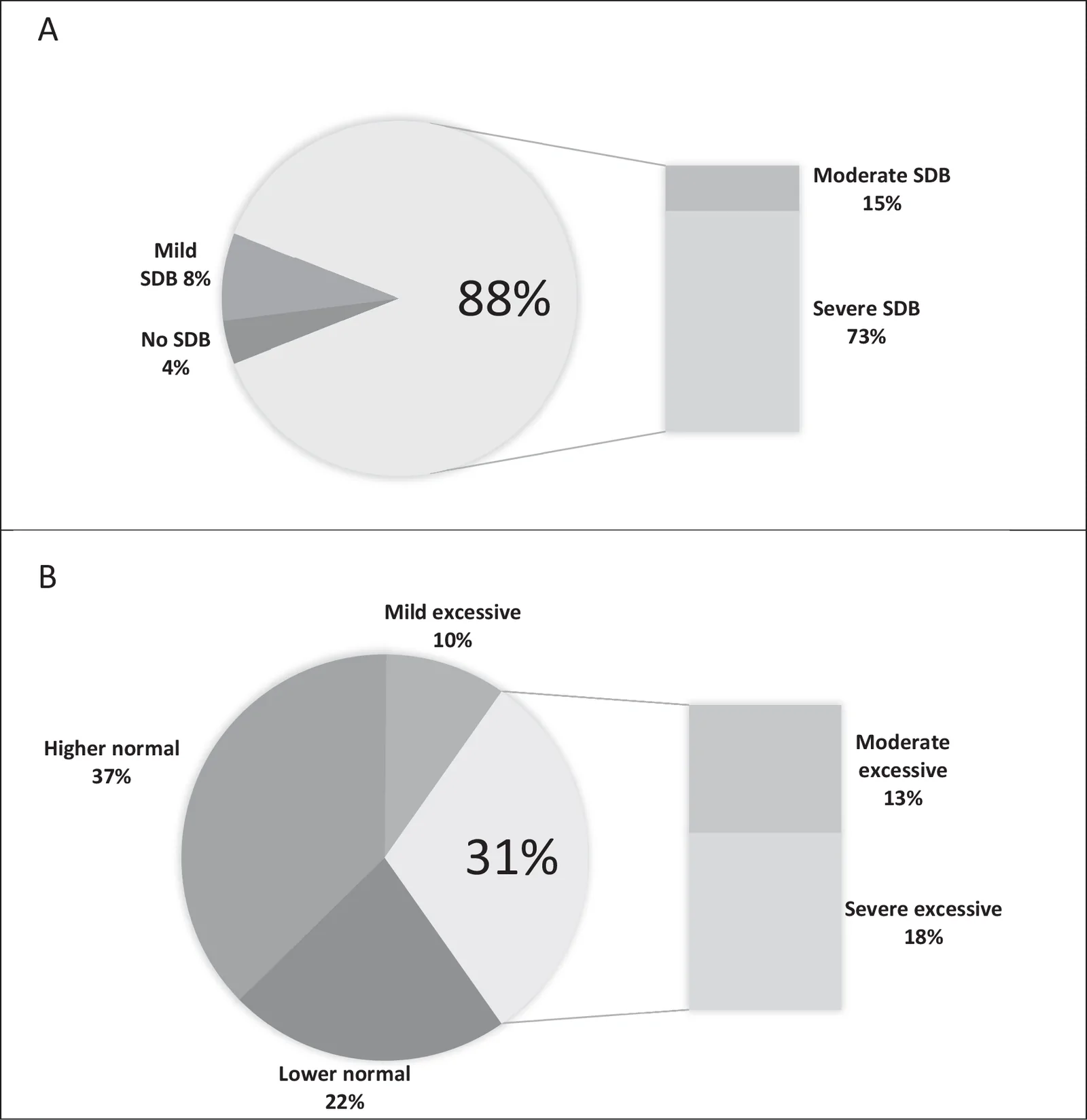
Sleep disordered breathing severity and daytime sleepiness distributions. Percentage of participants with sleep disordered breathing (SDB) severity classifications (**A**) and daytime sleepiness classification levels based on Epworth Sleepiness Scale (ESS) score (**B**). Moderate and severe SDB prevalence combined in one sector to indicate a sub-group that would warrant clinical treatment with nocturnal non-invasive ventilation.

Following 6 weeks of supervised RMT, PImax was significantly higher in the active group when compared with the sham group (Tables 2 and 3). The mean difference between the two groups (active-sham) was 11.8 cmH_2_O (95% CI: 5.2–18.4, *p* = 0.001). Based on severity classifications, 88% had moderate or severe SDB, and 8% had mild SDB. Ten percent of participants had mild daytime sleepiness while 31% reported moderate or excessive daytime sleepiness (Fig. 2).

**Table 2:** Outcome measures matched with sleep disordered breathing parameters at baseline, after 6-weeks of respiratory muscle training.

	Sham RMT group		Active RMT group		
	Baseline n = 26	6-Weeks n = 26	Baseline n = 22	6-Weeks n = 22	*P*-value 6-weeks
**Respiratory muscle strength measures**					
PImax (cmH_2_O)	50.0 ± 19.2	55.5 ± 21.4	50.6 ± 24.0	67.8 ± 23.5	0.001
PEmax (cm H_2_O)	32.0 ± 15.1	37.6 ± 19.2	33.0 ± 18.4	39.7 ± 23.6	0.775
**Lung function measures**					
FEV_1_ (l)	1.90 ± 0.73	1.89 ± 0.81	1.93 ± 0.79	2.00 ± 0.88	0.385
FVC (l)	2.50 ± 0.94	2.54 ± 1.04	2.55 ± 1.00	2.65 ± 1.11	0.349
Inspiratory Capacity (l)	2.06 ± 0.64	2.1 ± 0.8	2.13 ± 0.63	2.2 ± 0.7	0.546
Vital Capacity (l)	2.59 ± 0.94	2.7 ± 0.9	2.57 ± 0.89	2.8 ± 1.0	0.269
PEFc (l/s)	4.74 ± 1.71	4.87 ± 1.81	4.88 ± 1.34	5.01 ± 1.53	1.000
**Sleep disordered breathing parameters**					
AHI (events/h)	44 ± 25	40 ± 25	46 ± 21	45 ± 22	0.553
CSA (events/h)	1 ± 2	0 ± 1	0 ± 1	1 ± 3	0.173
OSA (events/h)	25 ± 20	23 ± 27	28 ± 23	26 ± 23	0.984
Hypopnoea (events/h)	16 ± 13	15 ± 10	14 ± 13	14 ± 12	0.937
REM AHI (events/h)	46 ± 19	39 ± 25	52 ± 31	45 ± 22	0.882
NREM AHI (events/h)	39 ± 25	36 ± 22	41 ± 21	42 ± 22	0.286
Min SpO_2_ (%)	74 ± 15	76 ± 10	76 ± 12	78 ± 10	0.756
Time SpO_2_ < 90% (%)	9 ± 13	12 ± 14	11 ± 13	11 ± 14	0.935
Mean SpO_2_ (%)	94 ± 2	93 ± 2	93 ± 2	94 ± 2	0.381
Sleep duration (mins)	286 ± 100	281 ± 97	285 ± 84	325 ± 84	0.079

Data shown as mean ± SD; *p* < 0.05 was considered significant for analysis of covariance adjusted for the baseline measures.

*RMT* respiratory muscle training, *PImax* maximal inspiratory pressure, *PEmax* maximal expiratory pressure, *FEV*_*1*_ forced expiratory volume in 1 s, *FVC* forced vital capacity, *PEFc* peak expiratory cough flow, *AHI* apnoea hypopnoea index, *CSA* central sleep apnoea, *OSA* obstructive sleep apnoea, *REM* rapid eye movement, *NREM* non-rapid eye movement, *Min SpO*_*2*_ minimum oxygen saturation; Time SpO_2_ < 90%, Percentage of time with oxygen saturation <90%; Mean SpO_2_, Mean oxygen saturation.

**Table 3:** Outcome measures matched with Epworth Sleepiness Scale at baseline, after 6-weeks of respiratory muscle training.

	Sham RMT group		Active RMT group		
	Baseline n = 32	6-Weeks n = 31	Baseline n = 30	6-Weeks n = 26	P-value 6-weeks
**Epworth Sleepiness Scale (ESS)**					
ESS total score (/24)	10.8 ± 6.2	10.1 ± 5.8	9.5 ± 5.7	9.2 ± 6.7	0.952
**Respiratory muscle strength measures**					
PImax (cmH_2_O)	51.5 ± 19.7	54.9 ± 21.3	48.4 ± 22.6	63.7 ± 24.0	<0.001
PEmax (cm H_2_O)	33.2 ± 14.0	37.4 ± 17.8	33.2 ± 14.0	37.4 ± 17.8	0.799
**Lung function measures**					
FEV_1_ (l)	1.90 ± 0.72	1.89 ± 0.78	1.76 ± 0.76	1.84 ± 0.85	0.385
FVC (l)	2.56 ± 0.93	2.55 ± 1.00	2.29 ± 1.00	2.46 ± 1.08	0.349
Inspiratory Capacity (l)	2.10 ± 0.69	2.11 ± 0.81	1.97 ± 0.72	1.99 ± 0.79	0.979
Vital Capacity (l)	2.58 ± 0.91	2.67 ± 1.07	2.3 ± 0.80	2.50 ± 0.99	0.126
PEFc (l/s)	4.66 ± 1.61	4.75 ± 1.76	4.56 ± 1.46	4.70 ± 1.55	0.893

Data shown as mean ± SD; p < 0.05 was considered significant for analysis of covariance adjusted for the baseline measures.

*RMT* respiratory muscle training, *PImax* maximal inspiratory pressure, *PEmax* maximal expiratory pressure, *FEV*_*1*_ forced expiratory volume in 1 s, *FVC* forced vital capacity, *PEFc* peak expiratory cough flow, *ESS* Epworth sleepiness scale.

**Fig. 3:**
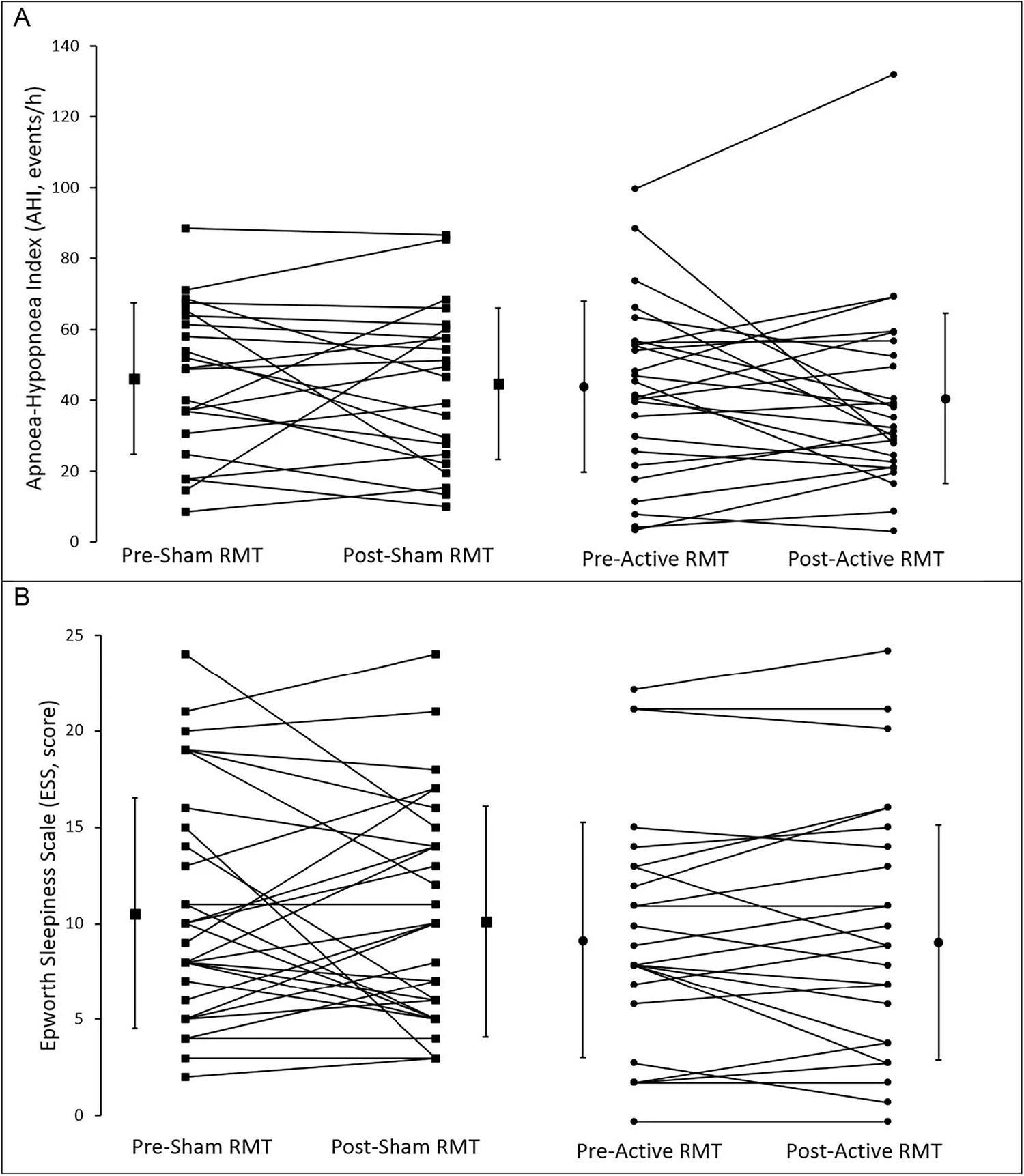
Individual participant Apnoea-Hypopnoea Index and Epworth Sleepiness Scale data. The Apnoea-Hypopnoea Index (AHI) and Epworth Sleepiness Scale (ESS) score data (**A** and **B**, respectively), pre- and post- 6-weeks of respiratory muscle training (RMT), individual participants are connected by a line. Participants in the Sham RMT group are represented by squares (left panels) and those in the Active RMT group by circles (right panels). Group means are represented by individual symbols and corresponding SD as a vertical line.

After 6 weeks of RMT there was no significant reduction in the AHI between the groups (active-sham), with a mean difference of 3 events/h (95% CI: −8–14, *p* = 0.553), nor was there a difference in ESS scores between groups (−0.06, 95% CI: −2.0–1.9, *p* = 0.952). Figure 3 shows individual AHI and ESS values (panel A and B, respectively) pre- and post- RMT for each participant, along with group means (±SD). There was no significant change in the remaining sleep or lung function parameters post training (Tables 2 and 3).

For people with acute tetraplegia, after 6 weeks of RMT there was no significant reduction in the AHI between the groups (active-sham), with a mean difference of 2 events/h (95% CI: −16–19, *p* = 0.845), nor was there a difference in ESS scores between groups (1.6, 95% CI: −3.3–6.4, *p* = 0.507). For people with chronic tetraplegia, after 6 weeks of RMT there was no significant reduction in the AHI between the groups (active-sham), with a mean difference of 3 events/h (95% CI: −13–18, *p* = 0.729), nor was there a difference in ESS scores between groups (−1.4, 95% CI: −3.6–0.9, *p* = 0.225). There was no correlation between baseline PImax or change in PImax and change in AHI (*p* = 0.397 and *p* = 0.847, respectively) or change in ESS (*p* = 0.219 and *p* = 0.199, respectively).

## Discussion

This study indicates that 6-weeks of intensive of RMT does not alter the severity of SDB nor reduce daytime sleepiness for people with tetraplegia. No between-group differences were observed between Sham-RMT and Active-RMT groups, despite a 23% greater increase in inspiratory muscle strength (PImax) in the Active-RMT group. The prevalence of SDB in our cohort was 96%, yet no between-group differences in SDB were found after RMT in the newly injured population nor in people with chronic tetraplegia.

Our results are similar to a 3-month RMT feasibility program in people with motor-incomplete SCI with no significant improvements in any outcome except PImax, for people with severe OSA [27]. Both studies are in contrast to a 12-week threshold-IMT program in people without SCI where the AHI and ESS were reduced by 6 and 20% for people with moderately severe OSA, with AHI scores <30 events/h being a predictor of responders [21]. However, our group predominantly had severe SDB, making direct comparisons difficult. Potentially our 6-week program in people with tetraplegia was not long enough to induce the changes seen in people without SCI. However, training occurred twice daily with improvements in PImax, similar to Sankari et al. [27], who did not see improvements in SDB measures or tongue strength [27]. While it is possible to measure the strength of some upper airway muscles, it was not the primary outcome of the parent study.

Night-to-night variability in sleep apnoea severity and the impact of single night testing on high misdiagnosis rates and neutral and inconsistent clinical trials findings is becoming increasingly recognised as a major issue for the field of sleep medicine [28, 29]. Indeed, night-to-night variability may be even more pronounced in the tetraplegic population [12, 30] due variable daytime fluid accumulation that can contribute to increased upper airway collapsibility [31] and autonomic instability. Thus, these variable influences may have contributed to, at least in part, a lack of between group difference in the current study.

The endotypic causes of OSA are multifactorial and vary between individuals [11]. RMT has the potential to decrease upper airway collapsibility by improving respiratory and upper airway muscle function. Future studies would benefit from inclusion of measures of genioglossus protrusion strength after RMT to determine if this could be a mechanism for improvement in SDB, or the investigation of strategies to improve respiratory muscle endurance as a therapeutic target. Future research with a larger sample size and longer training duration may provide a clinical treatment option with additional benefits to sleep duration, and carryover effects on quality of life and the performance of activities of daily living.

Similar to other studies [25, 27], and not unexpected in this analysis, was finding no change in lung function or expiratory muscle strength after 6-weeks of RMT. This could underlie the lack of change in SDB seen in this study. The RMT intensity at average ~50% PImax over 6-weeks was comparable to previous RMT studies in SCI [23, 24, 27], yet the absolute PImax of the cohort was relatively low (mean 50 cmH_2_O, ~40% predicted non-SCI). Thus, the absolute pressure at which the participants are training may be too low to effectively improve the strength of the upper airway muscles. This may be a limitation in this cohort because they are training their available intact respiratory muscles with a breathing effort that they perceived as severe (a modified Borg score of 5) [25]. Accordingly, in the absence of changes in lung function, RMT would not be expected to modify other OSA endotypes such as respiratory control stability (loop gain) during sleep. Similarly, changes in sleep drive that may influence the final OSA endotype, arousal threshold, would also not be anticipated with RMT.

In addition, RMT intensity may not be sufficient to overcome other physiological factors such as high nasal resistance. In people with tetraplegia and OSA, nasal resistance and resulting upper airway collapsibility during sleep is higher compared to non-SCI individuals with OSA [14] most likely due to sympathetic dysfunction and unopposed parasympathetic outflow. Nasal decongestants such as phenylephrine reduce nasal resistance but have no impact on the severity of SDB [32]. Although higher airway surface tension of the liquid/secretions of the upper airway mucosa in OSA results in increased airway collapsibility [33], surface tension in people with tetraplegia is not significantly different from non-SCI individuals [34]. While decreased lung volume may be a contributor to increased airway collapsibility due to decreased tracheal traction [13], we saw no changes in lung volume in sitting after RMT. Lung volumes were not measured supine during our study thus it cannot be determined if RMT affected supine lung volumes. Additionally, neither airway collapsibility, neck circumference nor abdominal girth were measured directly before and after RMT which are potential limitations of this study. Yet, RMT had no effect on the apnoea/hypopnoea ratio indicating that the upper airways remained highly collapsible regardless of training group.

Other studies that investigate the impact of muscle training on OSA in people without SCI have provided mixed results with the physiological mechanisms not clearly understood. In people without SCI, didgeridoo playing reduced the AHI and daytime sleepiness [35]; singing exercises reduced sleepiness and snoring [36]; and oropharyngeal exercises decreased neck circumference, snoring, daytime sleepiness and some people had reduced AHI [37]. Medications commonly used by people with tetraplegia (i.e., anti-spasticity, antidepressants, anxiolytics, which were kept stable during this study) may also contribute to daytime somnolence and mask potential RMT effects in this population.

The majority of our cohort had severe SDB, on average greater than 40 events/h with a large variance. To demonstrate a reduction of AHI by 15 events/h to change SDB severity category such that AHI is reduced to below 30 events/h, a sample of 46 participants per group would be required. Thus, in retrospect our study was underpowered to detect a clinically meaningful reduction in AHI, if present. However, this would appear unlikely given that the between group point estimates and 95% CIs were near identical in the current study. The lack of change in our study may also be related to the relatively short duration of RMT compared to the non-SCI population, where changes in SDB were observed after 12-weeks of RMT [20, 21]. Improvements seen in the case report and pilot studies are difficult to explain given our findings but may be related to the individual participant characteristics i.e. PImax, as some people were strong, or time since injury, as some people were still in the acute phase of SCI recovery [23, 24]. Additionally, in our sub-group analysis, the numbers of people in each group (acute/chronic and mild/moderate-severe) were small and not sufficient to demonstrate similar changes as seen in the non-SCI population. In the absence of evidence for RMT reducing SDB outcomes rehabilitation and future research should focus on improving compliance with and adherence to known SDB therapy (PAP). Improved sleep could lead to increased participation in physical rehabilitation programs and improved clinical outcomes, theorised to in turn reduce the autonomic, cardiovascular and motor co-morbidities associated with both tetraplegia and SDB [8].

RMT is a low-cost intervention that has been increasingly integrated into clinical care to increase inspiratory muscle strength after SCI. However, our study suggests that RMT at ~50% PImax does not reduce SDB or its consequences in people with acute or chronic tetraplegia over a short duration.

## Data Availability

Data are available from the corresponding author on reasonable request.
